# Aquatic Microcosms in Ecotoxicology: The Community-Level Ecological Risk Assessment of Pollutants

**DOI:** 10.3390/toxics13080694

**Published:** 2025-08-20

**Authors:** Dongning Yang, Yin Hou, Chao Wei, Jianan Ling, Xin Zheng

**Affiliations:** 1 State Key Laboratory of Environmental Criteria and Risk Assessment, Chinese Research Academy of Environmental Sciences, Beijing 100012, China; yangdongning24@mails.ucas.ac.cn (D.Y.); hy19909678451@163.com (Y.H.); weichao0524@163.com (C.W.); ling0jianan@163.com (J.L.); 2 Center of Eco-Environmental Monitoring and Scientific Research, Administration of Ecology and Environment of Haihe River Basin and Beihai Sea Area, Ministry of Ecology and Environment of People’s Republic of China, Tianjin 300170, China

**Keywords:** microcosm technology, standard aquatic microcosm, ecological effects, safety threshold

## Abstract

Microcosm technology serves as a sophisticated tool for simulating natural ecosystems, facilitating the examination of pollutants’ ecological impacts across population, community, and ecosystem scales. Currently, this technology finds extensive application in ecological toxicology and ecological risk assessment research. This concise review highlights the utility of microcosm technology in ecotoxicology, detailing the establishment of aquatic microcosms and analyzing key research trends to assess the ecological impacts of pollutants. It emphasizes the evaluation of pesticides, industrial chemicals, and heavy metals, providing a comparative analysis of safety thresholds derived from microcosm studies versus other methods. Finally, the review underscores the four urgent directions for future exploration: (a) track pollutant metabolites in microcosms; (b) develop microcosms with diverse species for natural ecosystem mimicry; (c) use DNA macrobarcoding to assess zooplankton and link it to species abundance; (d) study reasons behind no observed effect concentration (NOEC) vs. the 95% harmless concentration (HC5) values in microcosm studies. The determination of these directions helps to fill the gaps in understanding the fate and effects of pollutants within controlled ecosystem simulations.

## 1. Introduction

Anthropogenic activities are presently driving severe global environmental crises, with chemicals in consumer products posing a multitude of adverse effects on ecosystems and organisms. In numerous instances, these compounds exhibit carcinogenic and endocrine-disrupting properties, acting as toxic agents that impair individual organisms and induce physiological harm [1]. Moreover, many of these toxicants are capable of bioaccumulating in living tissues, leading to cascading adverse health outcomes over time [2]. Beyond threatening human health, these substances degrade soil integrity, pollute drinking water reservoirs, and disrupt terrestrial and aquatic ecosystems—harming both flora and fauna in the process [2]. A particularly pressing class of toxicants is emerging pollutants (EPs), a diverse group comprising pharmaceuticals (encompassing drugs and other products used in medicine and the pharmaceutical industry) [3], microplastics (polymers with various additives derived from the breakdown of larger plastic items or micro-plastic-containing products like cosmetics) [4], food-related chemicals (utilized in food production and preservation, such as shelf-life extenders, colorants, and flavorings), and agricultural chemicals along with their metabolic or transformation byproducts [5].

Currently, it is crucial to monitor and evaluate their impact on the environment and human health. Only in this way can measures be taken to regulate it and minimize the negative impacts. However, the pollution problem (especially pollution in the aquatic ecological environment) is still intensifying. The current monitoring and evaluation methods have not been fully studied and integrated, which limits the progress of researchers and policymakers in toxicological effects and risk assessment [6,7]. It is of great significance to conduct reliable scientific research on the evidence of these environmental problems and their risk impacts on the environment.

Microcosms are controllable biological models that simplify and simulate complex heterogeneous natural ecosystems. The core principle is that all units in nature, regardless of their size, exhibit similarities in structure and function [8]. As an experimental tool for simulating specific ecosystems, microcosms can be applied in laboratories or natural environments. They can be used to explore evolutionary and ecological processes on a short-time scale and are a key method in the field of ecotoxicology. They can be used to study the behavior of toxic substances and their impacts on the structure and function of ecosystems, as well as environmental remediation processes, and contribute to ecological risk assessment and environmental protection [9]. Through microcosms, researchers can not only collect quantitative data on phenomena like disruptions in energy and nutrient flows—aid in understanding the actual concentrations, half-lives, and degradation rates of toxic chemicals in the environment to support rational environmental risk assessment [10]—but also investigate ecosystem community structure and function, as well as screen the environmental impacts of toxic chemicals [11]. They can even simulate ecosystems inaccessible to humans, such as deserts, oceans, and volcanic craters. In the past, microcosms have contributed to the formation of important ecological concepts and are suitable for general ecological research and testing substances or organisms that are unsafe in natural environments. As an intermediate step between single-species toxicity testing and field research, their key feature is the introduction of species from multiple different taxonomic groups into the same test system, with models of varying complexity designable according to needs. They can not only be used to test biological toxicity but also to assess chemical fate, ecological interactions, and processes in other environmental components [12].

Based on the research object, the microcosm model can be divided into three categories: terrestrial microcosm, aquatic microcosm, and wetland microcosm [13], as shown in Table 1. The terrestrial microcosm is used to study the impact of pollutants on soil microbial communities. It typically contains components such as soil, plants, and soil microorganisms, and can simulate biological interactions and the effects of pollutants in natural environments under controlled conditions [14]. The wetland microcosm, also known as an artificial swamp system, is mainly used to study the persistence of pesticides in agricultural wetland environments and the migration and transformation of pollutants. It is also used to explore the possibilities and conditions for wetland systems to treat pollutants, as well as to understand the impact of surface runoff on aquatic ecosystems [15]. The aquatic microcosm is an experimental model that simulates aquatic ecosystems and is used to study the impact of pollutants on aquatic biological communities. It contains various aquatic organisms such as algae, protozoa, and crustaceans, as well as natural microbial communities [16]. The aquatic microcosm can simulate the basic functions and ecological processes of natural aquatic ecosystems. After a period of succession, the biological composition and metabolic activities can tend to be stable and have reproducibility. Both the wetland microcosm and aquatic microcosm are experimental models that simulate natural ecosystems and are used for ecological toxicology research. However, the wetland microcosm pays special attention to the wetland environment, including the interaction between aquatic and terrestrial organisms, while the aquatic microcosm focuses more on aquatic ecosystems, studying the impact of pollutants on aquatic biological communities.

In contrast, aquatic microcosms have broader research value, as their models’ biological compositions, nutrient cycles, and various physical parameters are very similar to those of natural ecosystems. They can be used to study fully functional ecosystems under controlled conditions, observe the interactions between multiple processes and components, and thus extrapolate the results more reasonably to natural ecosystems [17]. Aquatic microcosms can not only serve as a multi-species testing system for assessing chemical risks—especially at the population, community, and ecosystem levels—but also be used to reveal the ecological toxicity of pollutants and the degradation relationship between pollutants and environmental DNA (eDNA). This helps to comprehensively explore the migration and transformation laws of toxic pollutants, thereby extrapolating the results to natural ecosystems more reasonably.

However, the application of microcosms requires careful planning of resources, space, time scales, replication times, and similarity to natural ecosystems, and experimental and data processing must be integrated with mathematical models and field studies [1]. Nowadays, technologies for constructing more advanced microcosms in environmental research have been steadily evolving [18]. For example, the use of chemometric techniques in ecological risk assessment has been validated by multiple studies. Sakan et al. comprehensively evaluated the toxic element pollution status of the Vlasina River system (Serbia) using various chemometric methods, analyzing water and sediments from rivers in the Vlasina region [19]. Zhang et al. investigated the bioaccumulation of potentially toxic elements (PTEs) in soil–rice systems in the karst area of southwest China and assessed associated risks [20]. The results indicated that parent rock weathering and alluvial sediments are the primary sources of heavy metals in the soil, while fossil fuel combustion and agricultural activities also promote PTE accumulation in the soil. The high exceeding rates of Cd and Pb may be linked to their high bioaccumulation factors and contents in the soil. The average target hazard quotient (THQ) of PTEs in rice samples followed the order As > Cd > Cr > Cu > Zn > Pb > Hg. Therefore, health risks from excessive consumption of wild rice rich in heavy metals should be avoided. Lukić et al. reported the chemometric optimization of a solid-phase extraction (SPE) method for extracting 11 ultraviolet filters (UVFs) from urban lake water [21]. They employed the Plackett–Burman design, Box–Behnken design, and the Derrindzer desirability function to screen and optimize six SPE variables. Prior to determination by liquid chromatography-tandem mass spectrometry (LC-MS/MS), water samples were prepared using the optimized SPE method. Finally, Monte Carlo simulation and sensitivity analysis of environmental risks were conducted, revealing long-term safety concerns due to the persistent presence of UVFs.

In addition, to address limitations in microcosm applications, technologies for improving microcosms have been developed. For instance, in studies of root–soil interactions, natural soil’s high heterogeneity, variable and irreproducible microprocesses, opacity, and destructive sampling/operation pose challenges. While innovative methods like rhizobox systems [22] and microfabrication techniques exist [23,24], measurement tools remain inadequate—especially for dynamic processes or those influenced by pore structure. Model systems have thus become an effective strategy, making synthetic soil microcosms a key technological breakthrough; by constructing standardized soil simulation systems, they overcome the flaws of natural soil and provide a more controllable, reproducible experimental platform for studying microprocesses of root–soil interactions [25].

Therefore, this review first elaborates on the advanced technical means for constructing microcosms, and on this basis systematically clarifies the construction of standard aquatic microcosms. Subsequently, it separately explores specific application cases of microcosms in ecotoxicological research and their practical value in ecological risk assessment and conducts a comparative analysis with the research results of other methods. Through this systematic sorting and summarization, a strategic knowledge framework and theoretical support are provided for the in-depth development and application of microcosms in the fields of ecotoxicology and ecological risk assessment in the future.

**Table 1:** Classification of microcosm.

Main Types			Reference
**Terrestrial microcosm**	Terrestrial Microcosm System		[13]
	Root Microcosm System		[26]
	Soil Core Microcosm		[27]
	Soil Jar		[28]
**Aquatic microcosm**	Indoor	Aquaria	[29]
		Standardized Aquaria Microcosm	[30]
		Mixed Flask Culture Microcosm	[31]
	Outside	Steam Microcosm	[32,33,34]
		Pond and pool Microcosm	[35,36,37]
		Enclosed Column Microcosm	[38,39]
		Land-Based Marine Microcosm	[40]
		Reef and Benthic Microcosm	[41,42]
**Terrestrial (wetland) microcosm**	Simulate Farmland Ecosystem		[43,44,45]

## 2. Data Analysis Methods

**Figure 1:**
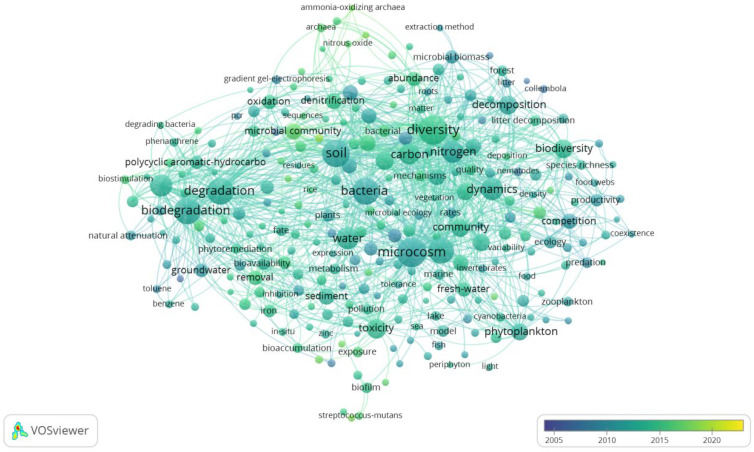
Labeled keywords in the field of microcosm technology from 2004 to 2024 (sampled from Web of Science). The size of the node represents the frequency of co-occurrence, and the color of the node corresponds to the chronological order of appearance.

In recent years, research on aquatic microcosms has significantly increased and has been widely applied in ecological studies. In order to obtain representative article samples, keyword searches were conducted using “microcosm technology” in the Web of Science Core Collection from 2004 to 2024. VOSviewer software (VOSviewer version 1.6.20) was used to perform evolutionary analysis on keywords related to microcosm technology from 2004 to 2024, targeting topics, titles, abstracts, and keyword fields in order to generate a keyword tag view (Figure 1). Frequent co-occurrence keywords include diversity, microcosm, soil, degradation, biodegradation, bacteria, growth, nitrogen, bioremediation, dynamics, water, toxicity, biodiversity, decomposition, and community. It is not difficult to see from the frequency of co-occurrence and the order of appearance time in Figure 1 that keywords related to aquatic microcosms account for the majority, and their association with ecotoxicology is much greater than that with soil microcosms.

## 3. Standardized Aquatic Microcosm

Due to the fact that the composition structure of aquatic microcosm models only includes the main components and ecological processes of natural ecosystems, compared with natural ecosystems, a microcosm, as a specific simulated ecosystem, has its structural and functional limitations. Moreover, aquatic microcosm simulated ecological models often rely on local organisms, water, and sediments, making it difficult to replicate and compare test results between different studies [46]. To address these issues, the establishment of a standard aquatic microcosm model involves constructing an artificial microcosm ecological simulation system using the same medium, sediment, and model organisms, which standardizes the research process and allows for more comparable results.

At present, a standard aquatic microcosm has been widely applied in ecological toxicology research to study the migration and fate of pollutants in environmental media. Radioactive tags are used to track the spatiotemporal distribution, migration, transformation processes, and transformation products of pollutants in the microcosm [10]. And they are also used to study the ecological toxicological effects of pollutants. Various types of microcosms have been used to study the adverse effects of pesticides and other pollutants on ecosystem structure and function, such as experimental laboratory cyclic aquatic microcosms, pond microcosms, aquatic terrestrial metaverses, and terrestrial metaverses [47]. Taub provided guidance for standardizing the composition and construction methods of aquatic microcosms, including culture media, biological components, species types, data processing, and statistical methods [48]. This standardized method allows for the repetition and reproduction of experimental results. By using standardized aquatic microcosms to study the effects of copper ions, it was found that the impact on biological communities is similar whether experiments are conducted in the same laboratory or in different laboratories.

The U.S. Environmental Protection Agency [49] established guidelines for mixed beaker microcosms and pond microcosms in 1996 [49]. The Organisation for Economic Co-operation and Development (OECD) released a method for constructing outdoor microcosms and mesocosms in 2006. In 2016, the American Society for Testing and Materials [50] developed the latest guidelines for constructing standardized freshwater microcosms [50]. The most commonly used guidelines are those provided by ASTM for building standardized aquatic microcosms. These guidelines specifically recommend 10 algal species (1 diatom, 2 cyanobacteria, and 8 green algae) and 5 invertebrate species (cladocerans, copepods, ostracods, rotifers, and protozoa) to establish the microcosm community (Table 2); for a detailed list of experimental designs for standard aquatic microcosm testing, refer to Appendix A. While standardized aquatic microcosms have less complexity compared to natural ecosystems, they still fulfill the requirements for observing ecosystem succession. However, due to the complexity and diversity of natural ecosystems, standardized aquatic microcosms are not able to satisfy the needs of different experimental purposes. Therefore, they can be optimized based on these guidelines, or specific aquatic microcosms can be developed to meet certain requirements.

## 4. Application of Aquatic Microcosm in Ecotoxicology Effects

### 4.1. Pesticides and Fungicides

Pesticides are usually stable organic toxic compounds, mainly used to control target organisms that affect crops [51]. However, only a small portion of the insecticides applied in farmland can act on the target objects, while most of the rest will spread into the environment [52]. This allows these compounds to enter environmental media such as soil, air, water, groundwater, or biota [53]. In addition, some insecticides can remain in the environmental media for a long time [54] and undergo bioaccumulation in organisms [53,55].

The toxic effects of various pesticides, especially chlorpyrifos, chlorothalonil, and isopropylnaphthalene, on aquatic ecosystems have been extensively studied at the Alterra Research Center of Wageningen University in the Netherlands [56]. These studies were conducted through indoor and outdoor microcosm experiments. The Center processed and analyzed the data obtained from the microcosm, and based on the observed short-term changes in community structure and the long-term resilience of microcosm, the researchers proposed grading criteria to describe the ecological effects. These criteria are shown in Table 3.

Chlorothalonil, a broad-spectrum pesticide, can interfere with cellular respiration by binding to glutathione [57]. In 2023, Iresha Sumudumali et al. established 18 microcosm systems (each containing 8 L of pond water) and set five Chlorothalonil concentration groups (0.010, 0.025, 0.100, 0.250, and 1.000 mg/L). After exposing plankton to the pesticide for 20 days, they conducted analyses using microscopic counting, chlorophyll a concentration measurement, and combined SIMPER (Similarity Percentage Analysis) with one-way ANOVA (Analysis of Variance) [58]. The results showed that among phytoplankton, *Dipterocarpus* (a genus of dipterocarp trees) and *Staurastrum* (desmid algae), as well as *Nudibranchus multispinellaris* (a nudibranch species) and nauplius copepods (zooplankton), were highly sensitive to Chlorothalonil. Under high Chlorothalonil concentrations, the abundance of some phytoplankton species increased, while trematodes exhibited tolerance. This study investigated the effects of Chlorothalonil on plankton community structure by simulating natural environments using microcosms. However, the experiment was designed only for short-term exposure, which limits its ability to fully reflect the long-term ecological effects of the pesticide.

Similarly, Suzie Kuyet Zaky et al. investigated the effects of atrazine exposure on plankton through an aquatic microcosm experiment [59]. Four treatment groups were set up in the experiment, the zooplankton-only group, the atrazine-only group, the combined group of both, and the control group, with an exposure time of 48 h. During this period, physical and chemical indicators such as temperature and dissolved oxygen were measured, the nutrient salt content was analyzed, and the Utermöhl method was used for the quantitative and taxonomic identification of phytoplankton. The results showed that atrazine had a negative impact on *Chlorophyta* but might promote the growth of diatoms and *gymnodinioids*. This study analyzed the responses of plankton from both taxonomic and morphological grouping perspectives, providing a multi-dimensional basis for the assessment of the ecological effects of atrazine.

However, the aforementioned aquatic microcosms were designed to focus solely on the short-term effects of a single pollutant, making it challenging to capture the cumulative and lag effects of pollutants. Cornejo, A. et al. explored the impacts of chlorpyrifos and chlorothalonil—both individually and in combination—on the survival and growth of detritivorous animals (*Anchytarsus*, *Hyalella*, *Lepidostoma*), as well as on the spore production rate, taxonomic richness, and community structure of aquatic hyphomycetes, using an aquatic microcosm experiment [60]. This study centered on the chronic toxic effects of the two pesticides and assessed their long-term ecological risks to freshwater plankton and other communities via microcosm simulation. A key focus was observing the temporal succession of plankton communities under sustained chlorothalonil exposure, analyzing whether communities could recover to a state near their initial condition or reach a new equilibrium within a specific period, thereby evaluating the resistance and resilience of freshwater plankton communities. The findings provide a basis for predicting long-term trends in natural freshwater ecosystems contaminated by chlorothalonil and offer scientific references for formulating environmental quality standards and setting risk early warning thresholds.

Beyond simulating the biotoxicity responses of pesticides under chronic exposure through the “long-term stability” of microcosms, Christian Villamarín et al. leveraged the “system integrity” of microcosms to link individual biological responses with ecological functions (leaf litter decomposition) and explore “multi-trophic level interaction effects.” [51] They focused on two pesticides (Engeo and chlorpyrifos) and investigated their impacts on aquatic organisms and related ecological processes via aquatic microcosm experiments. The results showed that under high-concentration (10 μg/L) chlorpyrifos exposure, the mortality of the trichopteran insect *Nectopsyche* sp. increased significantly, with a survival rate significantly lower than that of the control group (ANOVA and Tukey’s test indicated significant differences: diff = −1.39, *p* = 0.01). In contrast, the lethal effect of the same high concentration of Engeo was weak, showing no significant difference from the control group. Meanwhile, under both pesticide treatments, the survival rate of *Nectopsyche* sp. decreased significantly over time (R^2^ > 0.44 for low, medium, and high concentration groups, *p* < 2.2 × 10^−16^), indicating that toxic effects accumulated with exposure duration. In addition, the experiment indirectly reflected the responses of microorganisms and ecological functions by measuring the leaf litter decomposition rate of *Alnus acuminata* in the microcosms. It was found that the decomposition rate was mainly affected by time, dissolved oxygen, and pH, with no significant effect from pesticide treatments. This study enhanced the reference value of the results for natural ecosystems by simulating the natural environment of Andean rivers (e.g., 12 h light–dark cycle, 18 ± 1 °C temperature, and local gravel substrate) and controlling physicochemical conditions such as dissolved oxygen. However, it was limited to a single species, did not involve other species or interspecific interactions in the aquatic ecosystem, and failed to evaluate the cumulative effects of pesticides in sediment or their long-term impacts on benthic organisms.

Therefore, to delve deeper into mechanistic analysis, some researchers have introduced in vitro experiments and achieved cross-scale insights through the “microcosm + in vitro” integration. Lishani Wijewardene et al. conducted microcosm experiments using pond water from a nature reserve to investigate the toxic effects of two mixed herbicides: flufenacet and pyraclostrobin. The experiment included three concentration groups (0.5, 5, and 50 μg/L) and one control group, with a 28-day exposure period [61]. Using high-throughput sequencing and minimum inhibitory concentration [29] assays for aquatic bacteria, they found that, at 50 μg/L, both herbicides exhibited selective effects on aquatic bacterial communities; at 5 μg/L, only flufenacet showed such a selective effect. This study innovatively combined microcosm and in vitro experiments: it explored changes in microbial community diversity while analyzing direct effects on specific bacteria via in vitro assays, thereby revealing the interaction mechanisms between herbicides and aquatic microbial communities from multiple perspectives.

In addition to microcosmic applications in the study and risk assessment of various pesticides, microcosmic applications have also been applied to the study of common fungicides. Daniela Gómez-Martínez, et al. used microcosms to investigate how tebuconazole affects microbial biodiversity and whether it contributes to the release of transformation products from biofilms [62]. A combination of high-throughput sequencing (16S rRNA gene sequencing and ITS sequencing), multiple metrics (Shannon–Wiener index, Simpson index, etc.), and chromatography-mass spectrometry techniques showed significant changes in microbial biodiversity as the concentration of tebuconazole increased. The abundance of some sensitive microbial taxa decreased, while some resistant microorganisms may have relatively increased, leading to changes in microbial community structure. Meanwhile, specific transformation products were indeed released from the biofilm in the presence of tebuconazole, and their concentrations were correlated with tebuconazole concentration and time. This study revealed the complex effects of environmental concentrations of tebuconazole on microbial ecosystems and provided important data for assessing the ecological safety of tebuconazole. Unlike that study, Varghese, P. et al. focused more on how changes in triclosan concentrations acted on bacterial communities and enzyme activities in sediment ecosystems in their study of triclosan effects on microbial biodiversity, thus providing a scientific basis for assessing the ecological risk of triclosan [63]. Although both of them adopt similar microcosms and related technical tools, they have different focuses and thus provide different perspectives of environmental protection strategies for ecosystem risk assessment.

### 4.2. Fluorine-Containing Compounds

Florfenicol is an antibiotic widely used in aquaculture. Its mechanism of action is to bind to the A site of the 50S large subunit of the bacterial ribosome, interfering with the transpeptidation reaction of peptidyl transferase, thereby inhibiting bacterial protein synthesis. However, this drug has stable physical and chemical properties and is difficult to degrade. A large amount of unabsorbed florfenicol is continuously discharged into the environment, resulting in its persistent residue in the environment. This characteristic has raised extensive ecological concerns [64,65].

Numerous studies have used aquatic microcosms to explore the effects of florfenicol on aquatic organisms. For example, Zhang, T.Y. et al. focused on the structural changes of nirS-type denitrifying bacterial communities through microcosm experiments [66]. Given the central role of denitrification in the nitrogen cycle of aquatic ecosystems, they used this as an entry-point to evaluate the potential ecological risks of florfenicol. They mainly judged whether this drug would interfere with the nitrogen cycle process by affecting denitrifying bacterial communities, thereby having a negative impact on the function and stability of the entire aquatic ecosystem. In another study on nirS-type denitrifying bacteria, Zhang, T.Y. et al. further expanded the design of the microcosm experiment [67]. Based on the analysis of the impact of florfenicol on the structure of nirS-type denitrifying bacterial communities, they introduced other aquatic organisms. The aim was to explore the indirect effects of the structural changes of nirS-type denitrifying bacterial communities on other organisms and the interactions among organisms under the action of florfenicol. This design enables the study to more comprehensively reveal the relationship between florfenicol and the structure of nirS-type denitrifying bacterial communities, as well as its comprehensive ecological effects in the aquatic ecosystem.

In addition, Zhang et al. constructed an indoor aquatic microcosm [64]. By regulating the photoperiod and light intensity in the microcosm, they explored the interactive effects of these environmental factors and florfenicol on microorganisms in water and sediment. Based on the observed effects of florfenicol on resistance genes and bacterial community structure in water and sediment in the microcosm experiment, and considering the key role of microorganisms in the material cycle and energy flow of the aquatic ecosystem, the study comprehensively evaluated its environmental risks. The aim was to clarify whether florfenicol interferes with the normal functions and stability of the aquatic ecosystem by altering the microbial community structure and the distribution of resistance genes, providing a scientific basis for the rational use of florfenicol in aquaculture, the formulation of environmental management strategies, and ecological risk assessment.

All three studies revolved around the ecological effects of florfenicol. However, the research progressed from shallow to deep. It started by focusing on the structural level of a single microbial community, then extended to the functional level based on the study of microbial community structure. By introducing other organisms, it expanded to the interactions between organisms. Finally, taking the entire microbial community as the object and combining macroscopic ecological processes such as material cycling and energy flow, the coverage became more extensive. Correspondingly, the construction of the microcosm also changed accordingly. At first, it mainly served as a basic platform to simulate the natural aquatic environment for observing the structural responses of specific microbial communities. Then, multiple organisms were introduced to increase biological complexity, making it closer to the biological interaction relationships in natural ecosystems. Finally, by regulating environmental factors such as the photoperiod, the accuracy of simulating the natural environment was enhanced, and the analysis of multi-factor interactions was introduced. As a result, the application became more systematic, and it could more realistically reflect the ecological effects of florfenicol in the environment.

Per- and polyfluoroalkyl substances (PFAS) are persistent and ubiquitous organic pollutants in the environment [68,69,70]. Previous studies have been conducted at different locations, pollution levels, pollution types, and geochemical indicators, making it difficult to determine whether PFAS pollution directly affects indigenous microorganisms [71,72]. While laboratory experiments can control for specific environmental factors, there are challenges in modeling complex natural environments. Microcosm systems can control specific environmental parameters while simulating in situ conditions. Gang, D.G., et al. investigated the ecological risk of PFAS during phytoremediation through microcosm. Multigenerational studies were conducted using microcosm experiments to observe the effects of PFAS on the biological characteristics (e.g., growth, development, and reproduction) of key species across parental and offspring generations. These studies analyzed the accumulation and transmission patterns of PFAS across generations and their long-term impacts on population dynamics, and established dose–response relationships between PFAS concentrations and ecological risk indicators via microcosms. This provides a quantitative basis for assessing the ecological risks of PFAS in natural environments [73]. In addition to using microcosms to study the ecological risks of PFAS during phytoremediation, they can also be applied to investigate the toxic effects of PFAS on plants. Hanson, M.L., et al. set up different concentrations of perfluorooctanoic acid (PFOA) in a 12,000 L outdoor microcosm system and introduced two aquatic macrophytes (*Myriophyllum sibiricum* and *M. spicatum*) as research subjects into the microcosm. By measuring toxicity indicators such as the 10% effective concentration (EC_10_), 50% effective concentration (EC_50_), and NOEC, they assessed the toxic effects of PFOA on the growth and physiology of aquatic plants and determined the toxicity thresholds of PFOA for different aquatic plants after 14 to 35 days of exposure [74]. This study combined environmental fate data of PFOA with toxicity data on aquatic plants obtained from microcosm experiments to evaluate the toxic risk of PFOA to these aquatic plant species under current environmental concentrations. The conclusion was that the probability of these plants being exposed to PFOA under current environmental concentrations is negligible, providing a scientific basis for the ecological risk control of PFOA in the environment.

Microcosms can be applied not only to study the effect of PFAS on aquatic plants but also to study the community structure. Chao Guo set up four microcosm systems (river experiment, river blank, sea experiment, sea blank), set up PFOA-added and unadded controls, and sampled the data at multiple time points [75]. Combined with high performance liquid chromatography-tandem mass spectrometry (HPLC-TMS), CTAB, and high-throughput sequencing, the resulting data were then analyzed by a variety of statistical methods, including non-metric multidimensional scaling (NMDS), Spearman’s correlation analysis, and linear discriminant analysis of effect size (LEFSE). Ultimately, it was shown that the presence of PFOA had a significant effect on bacterial community structure and diversity. In addition, PFOA exposure led to functional changes in the bacterial community. This study also illustrates that microcosm experiments can more accurately model the actual effects of PFAS on natural bacterial communities than simple laboratory experiments, and that although effects on bacterial communities were only observed in the PFOA amended system, it contributes to the understanding of the effects of PFAS on ecosystems.

### 4.3. Hydrocarbon

Hydrocarbon pollutants are those substances in organic compounds composed of two elements, carbon and hydrogen, that cause harm to the environment and living organisms. Common hydrocarbon pollutants include methane, ethylene, aromatic hydrocarbons, etc., which are mainly derived from petroleum extraction and derivative industrial activities. Long-term exposure to such compounds not only causes damage to the human nervous system, respiratory system, etc., but also includes the risk of carcinogenicity and teratogenicity. Different natural environments were simulated by microcosm. Howland, K.E., et al. used a microcosm to simulate a freshwater coastal wetland environment and found that nutrient addition significantly enriched the hydrocarbon degradation potential of the microbial community by combining the microbial community analysis, hydrocarbon degradation efficiency assessment, and macro genomics technology [76]; Adebayo, O., et al. used a microcosm to simulate the deep-sea ecological environment system of the Northwest Atlantic Ocean by adopting an autoclave reactor to simulate the deep-sea high-pressure environment, while equipped with a refrigeration device to maintain low-temperature conditions (close to the actual water temperature of the deep sea), combined with microbial community analysis, hydrocarbon degradation efficiency assessment, and other techniques to reveal the characteristics and degradation mechanisms of hydrocarbon degrading microbial populations in the permanently cold deep-sea sediments in the Northwest Atlantic Ocean [77]. Using microcosm experiments to simulate different ecosystems, through the analysis and assessment of microbial communities and hydrocarbon degradation rates, researchers aim to understand the degradation rates of microbial communities degrading hydrocarbons in different natural environments, which provides a theoretical basis for understanding the carbon cycle and the natural degradation mechanisms of hydrocarbon pollutants in different ecosystems.

### 4.4. Microplastics

Microplastics [35] refer to plastic particles with a particle size of less than 5 mm, which originate from the weathering and decomposition of plastic waste. They are widely distributed in aquatic ecosystems, and their types are becoming increasingly diverse [78,79]. Eutrophic water bodies, significantly affected by human activities, have become sensitive areas for microplastic pollution. An increase in the content of microplastics in freshwater ecosystems can lead to water quality deterioration and interfere with the nitrogen cycle process [80]. Given the widespread presence and increasing diversity of microplastics, exploring their toxic effects in water environments has become an urgent research direction. In recent years, researchers have used aquatic microcosms to explore the toxic responses of aquatic organisms, especially aquatic microorganisms, to microplastics. The research has evolved from single processes to complex systems, from specific microbial groups to multi-trophic level associations, and from simplified controls to complex simulations, gradually approaching the authenticity of the natural environment.

Chang Tu et al. utilized the controllability of aquatic microcosms to isolate and analyze the effects of polystyrene nanoplastics (PS NPs) on the specific “plant-decomposer” ecological chain, clarifying the mechanism of microbial interactions [81]. Results showed that high concentrations of PS NPs significantly increased the diversity and abundance of bacteria and fungi, with key taxa involved in cellulose/hemicellulose decomposition—such as the fungal genera *Talaromyces* and *Fusarium*, and the bacterial genera *Sphingomonas* and *Aeromonas*—exhibiting a marked increase in abundance. This study is the first to reveal that elevated PS NP concentrations shift the bacterial–fungal relationship from antagonism to synergy, a transformation that serves as the core mechanism driving accelerated litter decomposition. It addresses the limitation of previous studies that focused solely on bacteria or fungi in isolation.

Similarly focusing on the advantages of microcosms in “mechanism elucidation”, Yu-Ting Shen et al. used aquatic microcosms to simulate pollution scenarios in farmland ditches, quantifying the interaction effects of conventional microplastics (polyethylene, PE), degradable microplastics (polylactic acid, PLA), and sulfonamide antibiotics (SAs) under individual and combined exposures, thus providing data for risk assessment. Results showed that *Acidobacteria* and *Proteobacteria* were the dominant phyla, accounting for 23.77–28.78% of the total [82]. Compared to the control group, microplastic treatments (PE, PLA) significantly altered the community structure (PCoA analysis, *p* < 0.05), and the interaction between SAs and microplastics induced non-additive effects (e.g., the community structure of the SA + PE group was more similar to that of the SA group than the PE group). In addition, PE and SA + PLA treatments significantly increased bacterial diversity, while the SA + PE treatment decreased it.

In the two typical studies mentioned above, the construction of aquatic microcosms focuses more on the local components of the ecosystem. However, there are also some studies that use aquatic microcosms to construct more complex systems, making the simulation of aquatic microcosms closer to the heterogeneity of the natural environment.

Yuanyuan Mo et al. integrated aquatic microcosms with environmental contexts to investigate the effect differences of tire wear particles (TWPs) along anthropogenic activity gradients, revealing “environmental dependency.” [83] Results showed that the impact of TWPs on aquatic microorganisms was stronger than on sediment microorganisms: In the water column, TWPs significantly altered bacterial composition (e.g., increased relative abundance of *cyanobacteria* in urban lakes and *Gammaproteobacteria* in rural lakes), while sediment bacterial communities remained largely unaffected. For fungi, TWPs significantly changed fungal composition in both the water column and sediments, with the magnitude of change in rural lakes being greater than that in urban lakes (e.g., decreased abundance of *Rozellomycota* and increased abundance of *Ascomycota* in both, but the changes were more pronounced in rural lakes).

Ze Hui Kong et al. maximized the system complexity of aquatic microcosms by introducing consumers (chironomid larvae) to elucidate the synergistic regulatory effects of biotic factors (consumers) and abiotic factors (microplastic properties), thereby more closely approximating the multi-factor interactions in natural ecosystems [84]. Results showed that low-concentration microplastics (1000 particles/kg) reduced the taxonomic richness of aquatic microbial communities; the effects of high-concentration microplastics (50,000 particles/kg) depended on the presence of chironomids: in the presence of chironomids, high-concentration microplastics significantly increased microbial abundance (+88.8%), whereas in their absence, abundance slightly decreased (−2.28%). In sediments, the presence of chironomids significantly reduced microbial diversity (−19.0%). Although the abundance of specific plastic-degrading bacteria (e.g., *Flavobacterium*, *Pseudomonas*) increased in microplastic-contaminated water, microbial community structure was primarily driven by habitat (water/sediment), time (June/July), and the presence or absence of chironomids—with the impact of microplastics being relatively weak.

Four classic studies have all relied on aquatic microcosms to reveal the ecological effects of microplastics, but they each have their own focuses in terms of research content. The first two studies demonstrate the advantages of microcosms in “mechanism analysis” (simplifying the system to focus on the core processes); the latter two studies show their potential in “complex system simulation” (integrating environmental heterogeneity and biological interactions to enhance the ecological relevance of the results). This difference not only reflects the development trend of microplastic research from basic mechanisms to comprehensive ecological risk assessment but also demonstrates the flexible adaptability of aquatic microcosms as a tool for different research objectives.

### 4.5. Metal Ion

Cadmium is a metal element commonly found in aquatic environments, which can accumulate in aquatic organisms, and its toxicity can be detected in aquatic organisms even at low concentrations [85]. Jiang et al. simulated the possible pollution and eutrophication in natural water bodies by setting up microcosms with different cadmium concentrations and nutrient levels. They observed the abundance and proportion of various groups in the plankton community and analyzed the changes in the abundance and proportion of various plankton such as green algae, diatoms, and zooplankton under different conditions, including high cadmium and low nutrient, and high cadmium and high nutrient, to clarify how nutrients enhance the response of the plankton community structure to cadmium [86]. Another study determined whether the dominant species of the plankton community would be replaced under the combined effect of cadmium and nutrients, as well as the rate and conditions of the replacement, for example, by studying whether the original dominant phytoplankton species would be replaced by others more pollution-tolerant or better adapted to eutrophication under eutrophic and cadmium-polluted environments by continuous monitoring of the microcosm [87].

In the research on cadmium, in addition to using microcosm experiments to study the structure of biological communities in the presence of cadmium pollution, microcosm experiments are also combined with chemical analysis techniques to detect the changes in the types and contents of metabolites produced by plankton under different cadmium concentrations and nutritional conditions. For example, the contents of metabolites such as toxins and organic secretions produced by zooplankton under different conditions of added nutrients and cadmium concentrations are analyzed to understand how nutrients affect the metabolic processes of plankton under cadmium stress [88]. In addition, microcosm technology can also be combined with methods such as metabolomics to study the changes in the metabolic pathways of plankton under the action of cadmium and nutrients, so as to determine which metabolic pathways are activated or inhibited. For example, determine the changes in pathways such as fatty acid metabolism and amino acid metabolism of plankton under high nutrient salts and cadmium pollution, so as to reveal the metabolic mechanism of plankton responding to cadmium under the condition of enhanced nutrient salts [89].

In addition, the absorption and accumulation of cadmium by plankton and the toxic effects of cadmium on the growth, reproduction, and physiological functions of plankton were studied in the microcosm. And at the same time, the presence of nutrients was observed to see whether they would affect the bioefficacy and toxicity of cadmium, for example, analyzing the content of cadmium in the body of the plankton and the differences in the effects of cadmium on the cellular structure of the plankton and the enzyme activities, etc., in different nutrient conditions [90]. Explore the pathways by which nutrients regulate the response of plankton to cadmium through microcosm technology. For example, determine whether nutrients change the tolerance and response degree of plankton to cadmium by affecting the cell membrane permeability and antioxidant system of plankton and further affect their ability to resist cadmium stress [91].

Through long-term microcosm experiments, monitor ecosystem indicators such as biodiversity, primary productivity, and material cycling rate; evaluate the impacts of cadmium and nutrients on ecosystem stability and function; and clarify the specific effects of the two on plankton and ecosystems based on the experimental results, so as to provide a scientific basis for the formulation of water pollution control strategies. Meanwhile, use microcosm experiments to evaluate the improvement effects of ecological restoration technologies such as the introduction of specific microorganisms and the addition of phytoremediation agents on plankton communities and water environments under the conditions of cadmium pollution and the presence of nutrients, and provide references for actual ecological restoration projects of water bodies.

## 5. Assessment of Ecological Risk Based on Aquatic Microcosm

As mentioned in the introduction, aquatic microcosm experiments can more effectively reflect the ecological effects of toxic substances, a feature that has made their application in evaluating pesticides and other toxic substances increasingly common. R. P. A. Van Wijngaarden et al. conducted a comparative analysis of studies published between 1980 and 2001 [92], which used aquatic microcosms to assess the ecological risks of two major neurotoxic insecticides (organophosphates and synthetic pyrethroids). The researchers standardized the thresholds for different studies using toxicity units and compared the microcosm-derived NOEC with the first-level methods (UP) in the EU management system. The results indicate that the NOEC from aquatic microcosms is more than 10 times higher than the EU level 1 acceptable concentration. This indicates that the threshold set by the EU for these two types of insecticides is sufficient for single organophosphate insecticides and has an excessive protective effect [93].

In another study by Geng Cuimin, the authors constructed a species sensitivity distribution (SSD) curve to determine the HC5 of the insecticide deltamethrin on algae (27.9 μg·L^−1^) and the HC5 of the herbicide butachlor on algae (9.3 μg·L^−1^) [94]. The results of standardized indoor aquatic microcosm experiments showed that the NOEC of deltamethrin and butachlor on algae were 10 μg·L^−1^ and 1 μg·L^−1^, respectively. Additionally, a study evaluated the sensitivity of freshwater organisms to Shirlan (with the active ingredient fluazinam) through single-species toxicity tests and microcosm experiments. The researchers constructed an SSD curve based on the acute toxicity data of 14 invertebrate species. The results showed that the HC5 obtained by the SSD method was 3.9 μg·L^−1^, while the NOEC obtained through the aquatic microcosm experiment was 2 μg·L^−1^. The responses of organisms to pollutants in the aquatic microcosm experiment were consistent with the results of the single-species tests, and the HC5 value calculated by the SSD method was slightly higher than the NOEC from the aquatic microcosm experiment.

In other studies, indoor aquatic microcosms have been used to investigate the effects of the fungicide triadimefon (a triazole fungicide, CAS: 43121-43-3) on freshwater planktonic communities. The results showed that its metabolite triadimenol (a triazole alcohol, CAS: 55219-65-3) had a more significant inhibitory effect on branchiopods, while triadimefon itself exerted a slight inhibitory effect on copepods at the beginning of the experiment [95]. During the entire experimental period, the NOEC of triazoles on planktonic animal communities was >2.08 × 10^3^ μg·L^−1^. The maximum residual concentration of triadimenol in water is 12.0 μg·L^−1^, which is significantly lower than the NOEC. This indicates that the risk of triazoles to planktonic animal communities is minimal. In further research, ecological risk assessment of the new fungicide azoxystrobin was conducted using outdoor microcosms and SSD methods [96]. The results of the two methods were compared, and it was suggested that the NOEC (0.33 μg·L^−1^) obtained through microsystems be used as a safety threshold for protecting aquatic organisms. In addition, Rodrigues et al. compared the microcosm and SSD methods for evaluating the ecological hazards of copper ions [97]. This study determined that the 63-day NOEC of Cu^2+^ in aquatic microcosm is 111 μg·L^−1^, and the HC5 (Hazardous Concentration for 5% of Species) obtained from the SSD curve is 3.28 μg·L^−1^. It is worth noting that the NOEC value was found to be higher than the HC5 value obtained by the SSD method. This difference can be attributed to the changes in Cu^2+^ bioavailability at different stages and interspecies feedback regulation in the aquatic microcosm.

Wang Zhen et al. conducted a study to evaluate the toxic effects of tetrabromobisphenol A on aquatic ecosystems [8]. The research results indicate that tetrabromobisphenol A has a more significant effect on the abundance of heteropods but has no significant effect on algae, large water fleas, and rotifers. In addition, researchers compared the NOEC obtained from aquatic microcosm experiments with the predicted no-effect concentration (PNEC) calculated using the SSD method. The NOEC in aquatic microcosm for 7–56 days is 10.80 μg·L^−1^, while the NOEC for 63 days is lower than that of azithromycin and significantly lower than the PNEC obtained by the SSD method (42.9 μg·L^−1^). This difference may be attributed to interspecies competition in the aquatic microcosm and interactions between different nutrient levels in different culture media [98,99]. Udo Hommen et al. used the SSD method recommended by the European Union, combined with the aquatic microcosm technology, to evaluate the safety threshold of the HC5 of nickel (Ni) in the aquatic ecosystem, and verified the effectiveness of this threshold in protecting the aquatic biological community [100]. Finally, the NOEC obtained from the aquatic microcosm was determined to be 12 mg Ni/L, approximately twice the HC5 value. This discovery indicates that the EU’s safety threshold based on HC5 effectively protects aquatic organisms.

The above experimental method uses an aquatic microcosm to conduct multi-species experiments to consider interspecific relationships and determine the NOEC at the population level. Then, the researchers compared this NOEC with the safety threshold derived from the SSD method to evaluate whether the threshold derived from SSD can adequately protect aquatic organisms. By adjusting the threshold, this method avoids “over-protection” and “under-protection” of aquatic organisms. As shown in Table 4, except for copper ions and nickel, the NOEC obtained from the microcosm experiment is generally lower than the HC5 of various substances derived from the SSD method. This indicates that simulating a mesoscale natural ecosystem under relatively realistic conditions can effectively determine the safety threshold for protecting aquatic organisms and contribute to the ecological risk assessment of pollutants. Compared with the SSD method, this is achieved by considering the impact of pollutants on the function and structure of the ecosystem and the interactions between species.

In addition, researchers have begun using aquatic microcosms to assess the toxicity of emerging pollutants. Ahmed et al. conducted a laboratory aquatic microcosm to evaluate the ecotoxicity of ciprofloxacin on small benthic nematode communities from Tunisia’s Seta Lagoon, revealing species-specific response mechanisms and abundance variations across ciprofloxacin concentrations [102]. Fluoxetine, as a selective serotonin reuptake inhibitor (SSRI), is widely used in the treatment of mental illnesses such as depression [103]. However, its toxic effects are not limited to the human body but also have significant impacts on the ecosystem. Even at low concentrations in water, fluoxetine may interfere with the growth and development, reproductive behavior, and neuroendocrine systems of fish and invertebrates. For example, it can affect the predation ability and reproductive success of fish, leading to a decline in population numbers; it can also change the metabolic rate and life cycle of invertebrates [104]. Moreover, when fluoxetine acts in combination with other pollutants (such as non-steroidal anti-inflammatory drugs and pesticides), it may produce synergistic or additive toxicity, exacerbating the damage to the aquatic ecosystem and affecting the stability of the food chain and ecological balance. Ji and Peng precisely controlled the concentrations of fluoxetine and ketoprofen through microcosm experiments to study their respective and combined ecotoxicological effects—for instance, observing whether the impacts of the mixed compounds on aquatic organisms in terms of growth, reproduction, and behavior differ from those of the single compounds [105]. Unlike other studies focusing on the ecotoxicity of single pollutants, this research employed microcosm experiments; by establishing single and mixed experimental groups for comparative tests, monitoring the growth and development of aquatic organisms, and integrating the cycling processes of carbon, nitrogen, phosphorus, and other substances in aquatic ecosystems, it not only explains the toxic effects of different pollutants both individually and in combination but also clarifies the processes and mechanisms of pollutant transformation and nutrient transfer to water bodies. It is increasingly regarded as an effective supplement to single or combined biotoxicity tests, strengthening the risk assessment framework for chemical substances and providing a basis for comprehensively and objectively understanding the aquatic ecosystem.

It is worth mentioning that Sisi Ye et al. conducted aquatic microcosm experiments on 367 phytoplankton samples in China and found that, due to the influence of exponential growth patterns, specific growth rate rankings show a linear distribution, resulting in an exponential distribution of relative phytoplankton biomass rankings [106]. Through mathematical derivation, it was revealed that the relative abundance and biomass rankings of phytoplankton exhibit an exponential distribution. Based on the distribution patterns of phytoplankton, this study derived these indices, which can be used for comprehensive analysis of phytoplankton communities and provide new insights for further evaluating the health of aquatic ecosystems.

## 6. Conclusions and Prospects

### 6.1. The Essence and Advantages of Microcosms

A microcosm is a controllable biological model that simplifies and mimics natural ecosystems. Its core principle is to study the ecological effects of pollutants in laboratory or natural settings by reproducing the key structures and functions of ecosystems. Based on the research objects, microcosms can be classified into terrestrial, aquatic, and wetland microcosms. Among them, aquatic microcosms are most widely used in ecotoxicological research because their biological composition, nutrient cycling, and physical parameters are highly similar to those of natural aquatic ecosystems, allowing for more reasonable extrapolation to natural systems. Their advantages lie in compensating for the deficiencies of single-species toxicity tests (which ignore inter-species interactions) and field studies (which involve complex environmental interferences) and serving as an “intermediate bridge” to reveal the comprehensive effects of pollutants at the population, community, and ecosystem scales.

### 6.2. Establishment and Specification of Standardized Aquatic Microcosms

To address the issue of incomparable results in traditional aquatic microcosms due to their reliance on local organisms and environmental media, international organizations (EPA, OECD, ASTM) have developed standardized guidelines. For example, ASTM recommends using 10 species of algae (such as *Anabaena cylindrica*, *Chlorella vulgaris*) and five species of invertebrates (such as *Daphnia magna*, *Hyalella azteca*) to construct communities, and standardizes parameters such as experimental containers, light cycles, and sampling frequencies (e.g., a 63-day test period, a 12 h light–dark cycle). Standardization significantly improves the reproducibility of experiments, makes the results from different laboratories comparable, and lays the foundation for cross-study analysis.

### 6.3. Application of Aquatic Microcosms in the Study of Ecotoxicological Effects

Aquatic microcosms have been widely used to assess the ecological effects of various pollutants. Traditional ecotoxicological studies rely on single-species tests (such as algal growth inhibition and daphnia mortality), but they ignore ecological processes such as interspecific competition, predation, and symbiosis, making it difficult to reflect the responses of real ecosystems. Aquatic microcosms achieve a leap from “individual toxicity” to “community function” by integrating multi-trophic level organisms (algae, invertebrates, microorganisms) and key ecological processes (material cycling, energy flow). For example, in pesticide research, microcosms not only observe the lethal effects of pollutants on single species but also reveal the “multi-trophic cascade reaction” in which pollutants indirectly inhibit litter decomposition by reducing detritivores (such as *Nectopsyche*), which cannot be captured by single-species tests.

### 6.4. Ecological Risk Assessment Value Based on Aquatic Microcosms

Aquatic microcosms provide key thresholds for ecological risk assessment by measuring the NOEC and complement the HC5 of methods such as SSD. This makes the establishment of safety thresholds more in line with reality and provides a scientific basis for environmental management.

### 6.5. Prospects

Currently, the application of aquatic microcosm in ecotoxicology and ecological assessment is far from being fully explored. In the future, the research scope can be expanded in various fields.

(a) In aquatic microcosm technology-based studies on pollutants, only pollutant concentrations are monitored, while their metabolites remain unmonitored. This represents a gap in the current understanding of the fate and effects of pollutants in microcosms. To comprehensively assess the environmental impact of pollutants, future studies should not only focus on the parent compounds but also include tracking of the metabolites. This will provide a more comprehensive understanding of the overall environmental effects of pollutants and their transformation products.
(b) The aquatic microcosms of most studies include algae, rotifers, *Daphnia magna*, and other zooplankton in neglecting benthos and microbial communities. As a result, the assessment of hazards caused by pollutant deposition in the sediment is inadequate. To enhance the population structure and function, it is necessary to construct an aquatic microcosm that encompasses a wider range of niche organisms.
(c) Study the direct toxicity and indirect effects of pollutants on various ecological groups in the aquatic microcosm, analyze the community effects and their mechanistic impacts of pollutants, and identify key environmental factors and pollutant-sensitive populations that contribute to community changes.
(d) Efficient, rapid, and accurate biological monitoring techniques, such as DNA macro barcode technology, will be employed to assess the composition of the zooplankton community in the aquatic microcosm. Additionally, the quantitative relationship between DNA macrobarcodes and species abundance in the aquatic microcosm are established.

Aquatic microcosm technology is commonly employed for studying the ecological hazard assessment of pollutants. Some studies have indicated that the NOEC aquatic microcosm exceeds the HC5 threshold. However, the underlying reasons for this disparity remain unclear and require further investigation. It is crucial to explore the factors that may contribute to this difference, such as the complexity of natural ecosystems compared to aquatic microcosms, the potential for compensatory responses within aquatic microcosms, or the influence of specific experimental conditions. Further research is necessary to elucidate these reasons and enhance the accuracy and reliability of microcosm technology in assessing the ecological hazards of pollutants.

## Figures and Tables

**Table 2:** Types, quantities, and introduction timings of organisms in Standardized Aquatic Microcosm Test Conditions.

Classification	Biological Species	All Kinds of Creatures Join the Microcosm Time/Day
Algae (initial concentration of 10^3^ cells/mL)	*Anabaena cylindrica*	0
	*Ankistrodesmus* sp.	0
	*Chlorella vulgaris*	0
	*Chlamydomonas reinhardi*	0
	*Lyngbya* sp.	0
	*Nitzschia kutzigiana*	0
	*Scenedesmus obliquus*	0
	*Selenastrum capricornutum*	0
	*Stigeoclontum* sp.	0
	*Ulothrix* sp.	0
Animals (depending on specific organisms)	*Daphnia magna* (16/microcosm)	4
	*Cypridopsis* (6/microcosm)	4
	*Philodina* sp. (0.03/mL)	4
	*Hyalella azteca* (16/microcosm)	4
	*Hypotrich* (0.1/mL)	4

**Table 3:** Classification standards of ecological effects of pollutants.

Effect Level		Description
I	No effect	The treatment group did not show any statistically significant effect, and there was no evident causal relationship between the treatment group and the control group.
II	Slight or temporary effect	The treatment group was observed individually to assess short-term effects.
III	Recoverable effect	An effect is observed; however, its duration does not exceed 8 weeks, as the system recovers within this timeframe following the final treatment.
IV	Definite effect	Although the immediate effect is evident, accurately estimating its long-term impact remains challenging.
V	Definite effect	The observed effect was evident, with a duration exceeding 8 weeks, and the system did not exhibit recovery within 8 weeks following the final treatment.

**Table 4:** Comparison between NOEC of different substances based on microcosmic test and other safety thresholds.

Substance	Exposure Mechanism	NOEC (μg·L^−1^)	Safety Threshold (μg·L^−1^)	Ratio (NOEC/Safety Threshold)
**Azinphos-methyl**	Single time	0.2	UP: 0.02	10
	Repeated	0.22	UP: 0.02	11
**Chlorpyrifos**	Single time	0.1	UP: 0.013	7.7
**Parathion**	Continuous	0.2	UP: 0.011	18
**Chlordecone**	Single time	5	UP: 0.33	15
**Parathion**	Single time	1.1	UP: 0.11	10
**Chlorpyrifos**	Single time	10	HC_5_: 27.9	0.36
**Butachlor**	Single time	1	HC_5_: 9.3	0.11
**Fluazinam**	Single time	2	HC_5_: 3.9	0.51
**Triadimefon**	Single time	>2.08 × 10^3^	Maximum residual concentration: 12.00	173
**Azoxystrobin**	Single time	0.33	HC_5_: 1.1	0.3
**Cu^2+^**	Single time	111	HC_5_: 3.278	33.8
**Ni**	Single time	1.2 × 10^4^	HC_5_: 4.2–6.8	1.76–2.86
**Tetrabromobisphenol A**	Single time	10.8	PNEC: 42.9	0.25

Note: UP is a first-tier acceptable concentration (EU, 1997) [101].

## Data Availability

No new data were created or analyzed in this study. Data sharing is not applicable to this article.

## Abbreviations

The following abbreviations are used in this manuscript:

eDNA	Environmental DNA
EPA	The U.S. Environmental Protection Agency
OECD	The Organisation for Economic Co-operation and Development
ASTM	The American Society for Testing and Materials
SIMPER	Similarity percentage program
NSAIDs	Nonsteroidal anti-inflammatory drug
PFAS	Per- and polyfluoroalkyl substances
HPLC–TMS	High-performance liquid chromatography-tandem mass spectrometry
NMDS	Non-metric multidimensional scaling
LEFSE	Linear discriminant analysis of effect size
SSD	Species sensitivity distribution
HC5	The 95% harmless concentration
PNEC	The predicted no-effect concentration
NOEC	The no observed effect concentration
SSRI	Selective serotonin reuptake inhibitor
TWPs	Tire wear particles
SAs	Sulfonamide antibiotics
PLA	Polylactic acid
PE	Polyethylene
PS NPs	Polystyrene nanoplastics
EC50	50% effective concentration
EC10	10% effective concentration
PFOA	Perfluorooctanoic acid
ANOVA	Analysis of Variance
LC-MS/MS	Liquid chromatography-tandem mass spectrometry
UVFs	Ultraviolet filters
SPE	Solid-phase extraction
THQ	Target hazard quotient
PTEs	Potentially toxic elements

## Appendix A

**Table A1:** Specific details of Standardized Aquatic Microcosm Test.

Projects	Biological Species/Test Requirements/Monitoring Tools	Classification	Test Requirements
**Experimental design**		Size and type of container	5 L glass jar, 16 cm in diameter, 25 cm in height, 10.6 cm in diameter
		Volume of culture medium	3 L
		Repetition number	6
		Concentration of experimental group	4
		Sampling frequency	Twice a week until the end
		Test cycle	63 d
**Physical and chemical parameters**	Temperature	20–25 °C	
	Workbench specification	>2.6 m × 0.85 m	
	Light quality	Cold/warm white light	
	Light intensity	79.2 µEM-2S-1PhAR	
	Light cycle	12 h light/12 h darkness	
	Culture medium	T82MV Culture medium	
	Sediments	200 g quartz sand, 0.5 g chitin and 0.5 g cellulose	
	pH	7.0 at the beginning	
**Test end point**	DO	Dissolved oxygen meter	
	pH	pH meter	
	Electric conductivity	Multi-parameter water quality analyzer	
	Turbidity	Turbidimeter	
	Algae density	Algae count box	
	Various zooplankton densities	Zooplankton counting box	
